# Comparison of outcomes for transjugular intrahepatic portosystemic shunt creation: Viatorr versus Fluency versus a bare stent/Fluency stent combination

**DOI:** 10.1186/s42155-024-00489-9

**Published:** 2024-10-18

**Authors:** Weizhi Li, Mengying Liu, Sheng Guan, Pengxu Ding, Jia Yuan, Yan Zhao, Peijie Li, Fuquan Ma, Hui Xue

**Affiliations:** 1 Department of Gastroenterology, First Affiliated Hospital of Xi’an Jiaotong University, 277 West Yanta Road, Xi’an, Shaanxi 710061 China; 2 Department of Vascular Surgery, People’s Hospital of Xinjiang Uygur Autonomous Region, Urumqi, China; 3 Department of Intervention, The First Affiliated Hospital of Zhengzhou University, Zhengzhou, China; 4 Department of Gastroenterology, Second Affiliated Hospital of Xi’an Jiaotong University, Xi’an, China

**Keywords:** Transjugular intrahepatic portosystemic shunt, Portal hypertension, Stent, Shunt dysfunction, Liver cirrhosis

## Abstract

**Purpose:**

To compare clinical outcomes of transjugular intrahepatic portosystemic shunt (TIPS) created with the single covered-uncovered stent (Viatorr TIPS Endoprosthesis) versus covered stent (Fluency) versus a combination of covered and uncovered stent.

**Materials and methods:**

From May 2016 and July 2019, a total of 180 liver cirrhosis patients with recurrent variceal bleeding underwent TIPS creation with single covered-uncovered stent (*n* = 63) or covered stent (*n* = 41) or a covered and uncovered stent combination (*n* = 76). Shunt dysfunction, rebleeding, overt hepatic encephalopathy and mortality was estimated using the Kaplan–Meier method and compared with the log-rank test.

**Results:**

The difference of baseline characteristics among these three groups were not significant. The included patients had a median age of 51 years (IQR 43–61), and 101 (56.1%) were men. The 1-year and 2-year shunt dysfunction rates were 1.6% and 3.2% in the single covered-uncovered stent group, 7.3% and 7.3% in the covered stent group and 5.3% and 6.6% in the combination group, respectively. There was no significant difference among groups [Hazard Ratio (HR) (95%CI): 1 vs 2.29 (0.38 − 13.72) vs 2.10 (0.41 − 10.83); *P* = 0.913]. No significant differences in the incidence of all-cause rebleeding were observed between the groups at 1 year (Viatorr vs Fluency vs combination: 11.1% vs 17.1% vs 10.5%) as well as 2 years (15.9% vs 17.1% vs 11.8%), with the HR (95%CI) being 1 vs 1.27 (0.5—3.21) vs 0.74 (0.30–1.82); *P* = 0.475). The 1-year and 2-year incidence of overt hepatic encephalopathy were 30.2% and 30.2% in the single covered-uncovered stent group, 22.0% and 22.0% in the covered stent group and 25.0% and 25.0% in the combination group, respectively. However, there was no significant difference among these three groups (*P* = 0.402). In addition, there was no significant difference in the 1-year and 2-year mortality (6.3% and 7.9% vs. 4.9% and 9.8% vs. 6.6% and 9.2%, *P* = 0.606).

**Conclusion:**

No significant difference was observed among different stent groups. Fluency covered stent and the generic bare stent/Fluency covered stent was not an unreasonable alternative to the Viatorr stent for TIPS creation.

**Supplementary Information:**

The online version contains supplementary material available at 10.1186/s42155-024-00489-9.

## Introduction

Transjugular intrahepatic portosystemic shunt (TIPS) represents a very effective treatment of complications of portal hypertension [1–3]. Shunt dysfunction is a major limitation when TIPS was created with bare metal stents, with dysfunction occurring in more than a half of cases after 1 year [4]. The availability of polytetrafluoroethylene (PTFE)-covered stent has dramatically reduced TIPS shunt stenosis related to intimal proliferation [5, 6]. Viatorr TIPS Endoprosthesis (W.L. Gore & Associates, Flagstaff, AZ, USA) are specifically designed covered stent grafts for TIPS placement. Although the Viatorr stent has been commercially available in the United States and European countries for two decades, PTFE–covered Fluency stent-grafts (Bard, Karlsruhe, Germany) had been the most widely used endoprosthesis for TIPS creation in China for a long time due to lack of the Viatorr stent-graft. The Viatorr and Fluency stent-graft are different in structures and product characteristics, the former has a 2-cm uncovered segment at the portal end and the latter has a 2-mm uncovered portion at each end [7].

In addition, Fluency stent has very high axial recoil which may lead to unbending and migration at its ends [8]. In order to overcome the shortcomings of Fluency stent mentioned above, some investigators have used a combination of bare mental stents and Fluency stents essentially mimicking the Viatorr stent design. A non-inferiority of a bare stent/Fluency covered stent combination to Viatorr stent was demonstrated [9–11]. However, there is still a shortage of comparative studies to compare the Viatorr stent with other stent-grafts for TIPS creation. Therefore, we conducted this retrospective multicenter study, aiming to compare shunt dysfunction and clinical outcomes of TIPS created with the specialized Viatorr stent versus single Fluency covered stent versus a bare stent/Fluency covered stent combination.

## Methods

### Study design and participant

In this retrospective multicenter cohort study, we extracted the data from a database of consecutive patients at four tertiary university hospitals between May 2016 and July 2019. The information of baseline characteristics and laboratory tests were collected. The need for informed consent was waived by the ethics committee due to its retrospective nature.

Patients were considered eligible for the study if they met the following criteria: liver cirrhosis (diagnosed by clinical presentations, laboratory tests, imaging findings or liver biopsies); age between 18 and 75 years; receiving elective TIPS for the first time to prevent variceal rebleeding. Exclusion criteria included: hemorrhage from ectopic varicose vein, a history of chronic or recurrent encephalopathy, Child–Pugh score > 13 points, hepatocellular carcinoma, portal vein thrombosis, creatinine greater than 3 mg/dL (265 μmol/L) and cardiac failure.

### Interventions

The TIPS creation was performed by a group of six highly experienced clinicians with more than ten years performing TIPS. There was no preference for the stent types among different operators. In clinical practice, the patients would be fully informed by the characteristics of different stents and its cost pre-operation. The selections of stents depended on consultation between the operators and patients as well as stent availability at time of procedure.

**Fig. 1:**
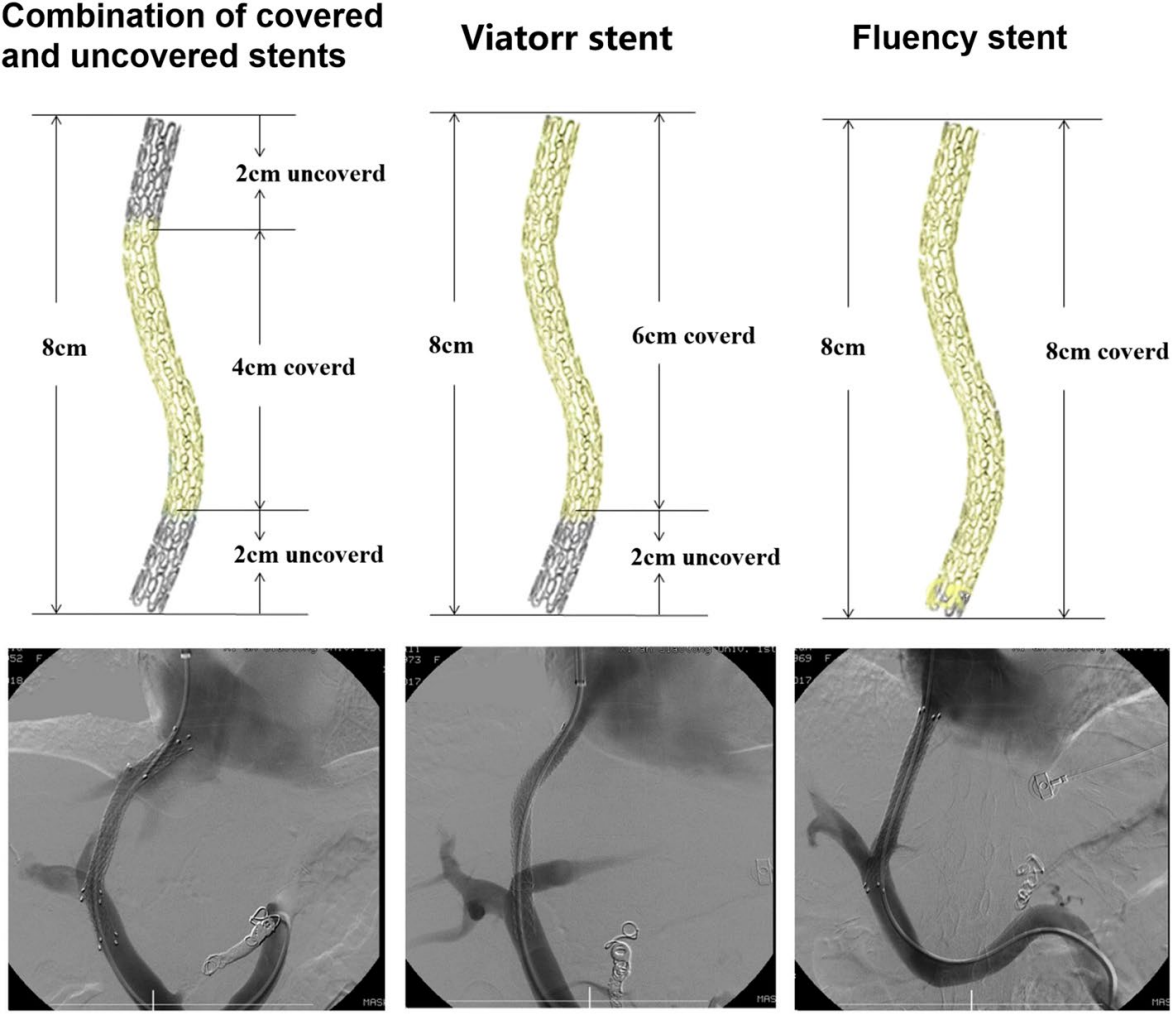
Transjugular intrahepatic portosystemic shunt (TIPS) creation with three different kinds of stents. The structure of the stents and venogram of post-operation were shown: (left) combination of the covered and uncovered stents; (middle) Viatorr stent and (right) Fluency stent

Patients were required to have computerized tomographic angiography of portal vein system to assist portal vein puncture. The operations were performed under local anesthesia at puncture site. Under the fluoroscopic guidance, an 18-gauge RUPS-100 needle (Rosh-Uchida TIPS set, Cook, Bloomington, IN) was introduced into the hepatic vein through a long 10-F vascular sheath inserted into the internal jugular vein and punctured through the liver parenchyma from the hepatic vein to the portal vein. A portal venogram was performed and the portacaval pressure gradient (PPG) was measured. The different stents structures and venogram were shown in Fig. 1.

For patients in the single covered-uncovered stent group, we chose an 8-mm Viatorr stent with 8 cm length that can extend from the portal vein access to the hepato-caval junction. For patients in the covered stent group, an 8 mm-diameter and 8 cm-length Fluency covered stent was deployed to connect the portal vein and the hepato-caval junction. For patients in the covered and uncovered stents combination group, an 8 mm in diameter covered Fluency stent with 4 cm in length was deployed first from the portal vein to the hepato-caval junction, followed by a longer 8 mm-diameter bare stent with 8 cm in length (Luminexx®, Bard, Karlsruhe, Germany) coaxially within it to cover the parenchymal tract, ie, to bridge from the portal vein to the hepato-caval junction. The distal uncovered portion of stents into the portal vein was about 2 cm and the proximal uncovered portion into inferior vena cava (IVC) was no more than 2 cm. All stents were dilated to 8 mm to obtain a good gradient (post-TIPS PPG lower than 12 mmHg or reduction more than 50%).

### Follow up

Follow-up were performed at patient visits to outpatient clinics, scheduled at 1 month, 3 months, and 6 months after TIPS creation and then every 6 months thereafter. At each visit, clinical, laboratory, abdominal ultrasound evaluations including shunt flow and velocity measurement in portal vein and intrahepatic branches were carried out and the information on prespecified liver-related complications that may have occurred since the previous visit were collected. In addition to scheduled assessment visits, angiography and PPG measurements were also performed if patients experienced variceal bleeding or ascites. Patients were followed until death, liver transplantation, or 1 year after the enrollment of the last patient.

### Endpoints and definitions

The primary outcome was shunt dysfunction, which was suspected when portal hypertensive complications (variceal bleeding or ascites) re-emerged or Doppler ultrasonography indicated shunt dysfunction (i.e., a reduction of portal blood flow velocity greater than 50% or below 28 cm/s, or a reversion of blood flow direction within the intrahepatic branches, or absence of flow within the shunt). However, shunt dysfunction was finally confirmed by portography and PPG measurements (PPG > 12 mmHg).

The secondary outcomes included all-cause rebleeding, overt hepatic encephalopathy (OHE) and death. Rebleeding was defined as recommended in the Baveno VI consensus [12]. Overt post-TIPS HE was defined as grade II or more HE according to the West-Haven criteria [13]. Large varices were defined as large or small if diameter ≥ 5 mm or < 5 mm, respectively [14, 15].

### Statistical analysis

Data are presented as median (interquartile range [IQR]) or number of patients and percentage values. Categorical and continuous variables were compared with the chi-square test and nonparametric Kruskal–Wallis test, respectively. Cumulative risks were estimated using the Kaplan–Meier method and compared with the log-rank test. Hazard ratios (HR) with 95% CI were calculated as an estimate of the effect size and its precision. Considering liver transplantation caused effect as a competing event on mortality, the post-hoc sensitivity analysis based on a competing risk approach was performed. The univariate and multivariate Cox regression analyses were performed to detect independent predictive factors for shunt dysfunction, recurrent bleeding, OHE and death. The variables included stent types, age, gender, etiology of cirrhosis, HBV-DNA detectable, ascites, Child–Pugh score, Child–Pugh class, Model for End-Stage Liver Disease (MELD) score, white blood cell, platelet count, bilirubin, albumin, international normalized ratio, creatinine, sodium, Pre-TIPS PPG, post-TIPS PPG and relative PPG reduction were entered into univariate analysis. Variables with *p* < 0.10 in univariate analyses were selected in the subsequent multivariate analysis. Correlated variables were not included together in order to reduce possible collinearities. The results are presented as subdistribution hazard ratio (sHR) with 95% confidence intervals (CI). Statistical significance was established at *P* < 0.05. All *P* values were two-tailed. All statistical calculations were performed using R 3.6.1 (http: //www.R-project.org/) software packages.

## Results

### Study patients

From May 2016 and July 2019, a total of 180 liver cirrhosis patients with recurrent variceal bleeding underwent TIPS creation with single covered-uncovered stent (*n* = 63) or covered stent (*n* = 41) or a covered and uncovered stent combination (*n* = 76). Table 1 gives baseline characteristics of these patients. The included patients had a median age of 51 years (IQR 43–61), and 101 (56%) were men and 79 (44%) were women. The main etiology of cirrhosis was chronic HBV infection (60%). The median MELD score was 10.3 points (IQR: 8.4—13.8) and the median Child–Pugh score was 7 points (IQR:6–8). In total, 123 (68%) patients underwent previous endoscopic variceal ligation treatment, and 128 (71%) patients underwent gastric variceal obturation treatment. 153 (85%) patients received oral Carvedilol treatment. Nine (5%) patients underwent previous splenectomy before TIPS creation. At baseline, 172 (96%) patients showed red-color sign (dilated small vessels or telangiectasia on the variceal surface on esophageal varices) under endoscopy examinations. The number of large esophageal varices were 57 patients (90%) in the Viatorr group, 37 patients (90%) in the Fluency group and 65 patients (86%) in the combination group (*p* = 0.695).

**Table 1:** Baseline characteristics

Variable	Viatorr (*n* = 63)	Fluency (*n* = 41)	Combination (*n* = 76)	*P* value
Age (years)	51.0 (44.0–59.0)	48.0 (40.0–62.0)	53.5 (44.5–62.0)	0.807
Gender, n (%)				0.572
Male	34 (54.0%)	21 (51.2%)	46 (60.5%)	
Female	29 (46.0%)	20 (48.8%)	30 (39.5%)	
Etiology of cirrhosis, n (%)				0.586
Hepatitis B virus infection	40 (63.5%)	22 (53.7%)	46 (60.5%)	
Hepatitis C virus infection	5 (7.9%)	5 (12.2%)	3 (3.9%)	
Alcoholic liver disease	4 (6.3%)	1 (2.4%)	3 (3.9%)	
Autoimmune hepatitis	3 (4.8%)	0 (0.0%)	2 (2.6%)	
Primary biliary cholangitis	4 (6.3%)	4 (9.8%)	7 (9.2%)	
Cryptogenic	7 (11.1%)	9 (22.0%)	15 (19.7%)	
HBV-DNA detectable, n (%)	10 (15.9%)	7 (17.1%)	11 (14.5%)	0.930
Previous encephalopathy, n (%)	3 (4.8%)	4 (9.8%)	3 (3.9%)	0.406
Location of varices				0.789
Esophageal varices only	12	7	17	
Esophageal and gastric varices	51	34	59	
Size of varices				0.695
Small varices	6	4	11	
Large varices	57	37	65	
Ascites, n (%)	42 (66.7%)	27 (65.9%)	47 (61.8%)	0.820
Child–Pugh score (points)	8 (6–8)	7 (6–8)	7 (6–8)	0.428
Child–Pugh class, n (%)				0.776
A	19 (30.2%)	12 (29.3%)	23 (30.3%)	
B	41 (65.1%)	27 (65.9%)	52 (68.4%)	
C	3 (4.8%)	2 (4.9%)	1 (1.3%)	
MELD score (points)	10.3 (8.4–12.1)	10.1 (9.0–11.7)	10.4 (8.8–11.8)	0.852
White blood cell (× 10^9^/L)	2.5 (1.9–3.4)	2.6 (1.8–3.6)	2.5 (1.8–3.8)	0.995
Haemoglobin (g/L)	84.0 (74.5–97.5)	81.0 (71.0–87.0)	80.0 (74.0–93.0)	0.477
Platelet count (× 10^9^/L)	64.0 (41.0–87.5)	56.0 (45.0–83.0)	64.0 (38.5–83.5)	0.982
International normalized ratio	1.3 (1.2–1.4)	1.3 (1.2–1.4)	1.3 (1.2–1.4)	0.410
Alanine aminotransferase (U/L)	23.5 (15.2–30.0)	21.0 (14.0–26.0)	26.0 (17.0–37.0)	0.042
Aspartate aminotransferase (U/L)	29.0 (20.0–35.5)	27.0 (19.0–32.0)	30.0 (24.0–39.5)	0.088
Albumin (g/L)	33.4 (30.6–35.7)	34.3 (31.9–37.4)	34.5 (31.6–38.2)	0.177
Bilirubin (μmol/L)	20.6 (13.8–27.6)	21.6 (16.2–25.6)	22.8 (15.7–31.8)	0.314
Blood urea nitrogen (mmol/L)	4.7 (3.6–6.4)	4.5 (3.4–5.8)	5.7 (4.1–7.1)	0.156
Creatinine (mg/dL)	0.7 (0.5–0.8)	0.6 (0.5–0.7)	0.7 (0.6–0.8)	0.196
Sodium (mmol/L)	140.0 (138.0–143.0)	142.0 (140.0–143.0)	140.0 (139.0–143.0)	0.180
Pre-TIPS PPG (mmHg)	24.0 (21.0–27.0)	24.0 (20.8–26.0)	23.0 (20.0–26.0)	0.500
Post-TIPS PPG (mmHg)	12.0 (9.0–14.5)	9.0 (6.0–10.2)	10.0 (7.0–12.0)	< 0.001
Absolute PPG reduction (mmHg)	12.0 (9.5–14.0)	15.0 (11.0–17.8)	13.0 (10.5–15.5)	0.039
Relative PPG reduction (%)	52.0 (42.2–59.1)	61.1 (52.3–72.8)	54.2 (47.3–67.8)	0.003

Data are median (IQR), or n (%)

*HBV* Hepatitis B virus, *MELD* Model for End-Stage Liver Disease, *PPG* Portacaval pressure gradient, *TIPS* Transjugular intrahepatic portosystemic shunt

**Fig. 2:**
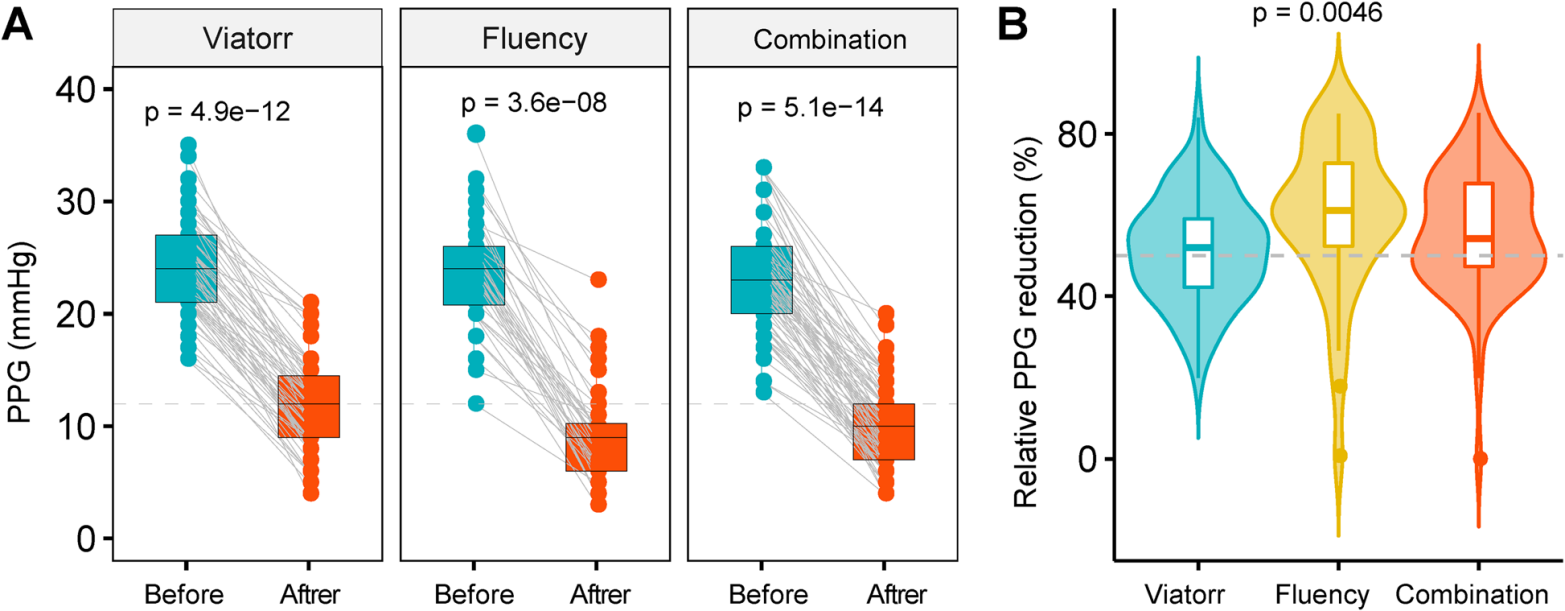
Portacaval pressure gradient changes after TIPS placement. **A** Paired comparison of pre-TIPS and post-TIPS PPG in each group; (**B**) The violin plot showed the comparison of relative pressure decrease in the three groups after TIPS placement. Abbreviations: PPG, portacaval pressure gradient; TIPS, transjugular intrahepatic portosystemic shunt

TIPS procedures were successfully performed in all patients. Most of the patients (95%) underwent TIPS treatment via the left portal vein (*n* = 171) in order to decrease the postoperative hepatic encephalopathy and shunt dysfunction. In Viatorr group, two of 63 patients (3%) underwent TIPS creation with one Viatorr stent and one bare stent as an extension stent to the hepatic vein/IVC junction. In Fluency group, one of 41 patients (2%) underwent TIPS creation with two Fluency stents as extension. The mean SD PPG decreased immediately after stent placement in three arms, from 24.3 ± 4.4 to 11.9 ± 4.0 mmHg in Viatorr group, from 23.8 ± 4.9 to 9.0 ± 4.2 mmHg in Fluency group and from 23.3 ± 4.8 to 10.0 ± 3.6 mmHg in combination group (*p* < 0.001) (Fig. 2A). The violin plot showed that the relative reduction of PPG was greater in the Fluency groups (51% ± 13% vs 60% ± 18% vs 57% ± 13%, respectively, *p* = 0.010) (Fig. 2B).

Follow-up ended on July 1, 2020. Two patients in the combination stent group received liver transplantation. No patients were lost to follow up. The median follow-up was 26.0 months (IQR: 19.9–37.7).

### Shunt dysfunction

Two patients (3%) in the Viatorr group, 3 patients (7%) in the Fluency group and 5 patients (7%) in the combination group developed shunt dysfunction (*p* = 0.597) (Table 2). The first episode of shunt dysfunction was suspected according to the routine follow-up ultrasound in 1 (2%) patient, 0 (0%) patient, 1 (1%) patient in the Viatorr, Fluency, combination group respectively; and according to recurrence of portal hypertension-related complications in 1 (2%), 3 (7%), 4 (5%) in the Viatorr, Fluency, combination group respectively. The 1-year and 2-year shunt dysfunction rate were 2% and 3% in the Viatorr group, 7% and 7% in the Fluency group and 5% and 7% in the combination group, respectively. There were no significant differences among groups (HR [95%CI]: 1 vs 2.29 [0.38 − 13.72] vs 2.10 [0.41 − 10.83]; Log-rank test: *p* = 0.913; Supplementary Fig. 1A). Shunt dysfunction rate also did not significantly differ in competing risk analysis (sHR [95%CI]: 1 vs 2.35 [0.40 − 13.97] vs 2.09 [0.41 − 10.69]; Gray’s test: *p* = 0.587; Supplementary Fig. 2A). On univariate and multivariate Cox regression analysis, gender was the only variable marginally associated with shunt dysfunction (Supplementary Fig. 3 and Fig. 4A).

**Table 2:** Summary of outcome measurements

Outcome	Viatorr (*n* = 63)	Fluency (*n* = 41)	Combination (*n* = 76)	*P* value
Median duration of follow-up (months)	25.0 (19.6–34.3)	29.0 (22.6–41.3)	27.0 (18.3–37.9)	0.072
**Shunt dysfunction**				
Number of patients (%)	2 (3.2%)	3 (7.3%)	5 (6.6%)	0.597
Clue of shunt dysfunction, n (%)				0.580
Ultrasound	1 (1.6%)	0 (0.0%)	1 (1.3%)	
Symptom recurrence	1 (1.6%)	3 (7.3%)	4 (5.3%)	
**Rebleeding**				
Number of patients (%)	10 (15.9%)	8 (19.5%)	9 (11.8%)	0.526
Sources of bleeding, n (%)				0.938
Variceal rebleeding	7 (11.1%)	6 (14.6%)	7 (9.2%)	
Portal hypertensive gastropathy	2 (3.2%)	1 (2.4%)	1 (1.3%)	
Peptic ulcer bleeding	1 (1.6%)	1 (2.4%)	1 (1.3%)	
Rebleeding treatment				0.239
TIPS revision	6 (9.5%)	7 (17.1%)	5 (6.6%)	
Drug or endoscopy	4 (6.3%)	1 (2.4%)	4 (5.3%)	
**Overt hepatic encephalopathy**				
Number of patients (%)	20 (31.7%)	9 (22.0%)	19 (25.0%)	0.495
Spontaneous overt HE, n (%)	15 (23.8%)	6 (14.6%)	17 (22.4%)	0.048
Precipitating overt HE, n (%)				
Precipitating event, n (%)				
Constipation	3 (4.8%)	0 (0.0%)	0 (0.0%)	
Gastrointestinal bleeding	1 (1.6%)	0 (0.0%)	2 (2.6%)	
Higher protein intake	1 (1.6%)	0 (0.0%)	0 (0.0%)	
Infection	0 (0.0%)	3 (7.3%)	0 (0.0%)	
Severe HE	6 (9.5%)	3 (7.3%)	5 (6.6%)	0.882
Refractory HE	0 (0.0%)	0 (0.0%)	4 (5.3%)	0.082
**Death**				
Number of patients (%)	5 (7.9%)	6 (14.6%)	7 (9.2%)	0.562
Cause of death, n (%)				0.678
Gastrointestinal bleeding	0 (0.0%)	1 (2.4%)	1 (1.3%)	
Liver failure	3 (4.8%)	4 (9.8%)	3 (3.9%)	
Multiorgan failure	1 (1.6%)	0 (0.0%)	0 (0.0%)	
Unrelated to liver disease	1 (1.6%)	1 (2.4%)	3 (3.9%)	
**Liver transplantation**, n (%)	0 (0.0%)	0 (0.0%)	2 (2.6%)	0.509
**Hepatocellular carcinoma**, n (%)	3 (4.8%)	3 (7.3%)	1 (1.3%)	0.186

Data are n (%),

*HE* Hepatic encephalopathy

### Rebleeding

Ten patients (16%) in the Viatorr group, 8 patients (20%) in the Fluency group and 9 patients (12%) in the combination group experienced rebleeding during follow-up (Table 2). In 7 (11%) patients from the Viatorr group, 6 (15%) patients from the Fluency group and 7 (9%) patients from the combination group, the recurrent bleeding was due to varices. In the Viatorr group, 6 patients underwent TIPS revision and 4 patients received medical or endoscopic treatment as primary therapy for management of rebleeding. The corresponding numbers of patients were 7 and 1 in the Fluency group and 5 and 4 in the combination group, respectively. No significant differences in the incidence of all-cause rebleeding were observed between the groups at 1 year (Viatorr vs Fluency vs combination: 11% vs 17% vs 11%) as well as 2 years (16% vs 17% vs 12%), with the HR (95%CI) being 1 vs 1.27 (0.5—3.21) vs 0.74 (0.30–1.82); Log-rank test: *p* = 0.475, Supplementary Fig. 1B). Similar results were obtained in competing risk test (sHR: 1 vs 1.32 [0.52–3.32] vs 0.74 [0.31–1.79]; Gray’s test, *p* = 0.498, Supplementary Fig. 2B). On univariate and multivariate Cox regression analysis, MELD score was the only variable marginally associated with all-cause rebleeding (Supplementary Fig. 5 and Fig. 4B).

### Overt hepatic encephalopathy

Twenty patients (32%) in the Viatorr group, 9 patients (22%) in the Fluency group and 19 patients (25%) in the combination group developed at least one episode of OHE during follow-up (Table 2). Among them, 6 patients (16%) in the Viatorr group, 3 patients (7%) in the Fluency group and 5 patients (7%) in the combination group developed severe HE (grade III/IV). In the Viatorr and Fluency groups, encephalopathy was successfully controlled medically in all patients. In the combination group, however, 4 patients presented with refectory encephalopathy and were treated by shunt reduction. The 1-year and 2-year OHE rate were 30% and 30% in the Viatorr group, 22% and 22% in the Fluency group and 25% and 25% in the combination group, respectively. However, there was no significantly statistical differences among these three groups (HR [95%CI]: 1 vs 0.67 [0.30 − 1.48] vs 0.76 [0.41 − 1.43]; Log-rank test: *p* = 0.402; Supplementary Fig. 1C). The OHE rate also did not significantly differ in competing risk analysis (sHR [95%CI]: 1 vs 0.69 [0.32 − 1.49] vs 0.77 [0.42 − 1.41]; Gray’s test: *p* = 0.558; Supplementary Fig. 2C). Univariate analysis and multivariate Cox proportional hazard model showed that increased age and creatinine levels were marginally associated with OHE (Supplementary Fig. 6 and Fig. 4C).

### Mortality

The causes of death were summarized in Table 2. There were no significant differences in the 1 year and 2 year mortality (Viatorr: 6% and 8% vs Fluency: 5% and 10% vs combination: 7% and 9%, Supplementary Fig. 1D), with the HR being 1 vs 1.70 [0.52 − 5.58] vs 1.12 [0.35 − 3.52]; Log-rank test: *p* = 0.606. Similar results were observed when using competing risk analysis (sHR [95%CI]: 1 vs 1.84 [0.57 − 5.99] vs 1.16 [0.37 − 3.65]; Gray’s test: *p* = 0.547; Supplementary Fig. 2D). Univariate and multivariable analysis showed that age was independent predictors of death (Supplementary Fig. 7 and Fig. 4D).

### Change of liver function

As shown in Supplementary Fig. 8, the changes of liver function were not significantly different among groups.

### Economic analysis

Follow-up economic data could be obtained for 123 patients: 39 in the Viatorr group 33 in the Fluency group and 51 in the combination group. The cost comparisons revealed an average cost per patient of 154.8 ± 15.9 k¥ in the Viatorr group vs. 172.5 ± 170.8 k¥ in the Fluency group vs. 158.5 ± 77.9 k¥ in the combination group, showing no significant difference (*p* = 0.775).

## Discussion

In this multicenter retrospective study, we showed that covered Fluency stent and the generic bare/covered Fluency stent combination was not inferior to specialized Viatorr covered stent for TIPS creation. The Fluency covered stent alone and the bare stent/Fluency covered stent combination had a shunt patency comparable with that of TIPSs created by Viatorr devices, with similar rate of rebleeding, hepatic encephalopathy and death. To the best of our knowledge, this is the first controlled study comparing these three types of stents in terms of the examination of shunt dysfunction and clinical efficacy.

The 1-year TIPS patency rates in our study were favorable and comparable to that of TIPSs created by covered stent-grafts in the literature (76%–95%) [4–6, 9, 16, 17]. Furthermore, the shunt patency rates were not different among the groups. This result was consistent with that in a previous RCT comparing bare stent/Fluency covered stent combination with single Fluency covered stent, where the two techniques were comparable in the primary shunt patency [9]. However, it was contrast with an earlier study by Saad et al. showing that the Viatorr stent was associated with improved patency compared with the bare/Fluency stent-graft combination [10]. There were several differences between these two studies. Firstly, in Saad’s study, the combination group used a different kind of bare stent (Wallstent; Boston Scientific, Natick, Massachusetts) and the sample size in the combination group was relatively small with only 28 patients. Secondly, in the former study the bare stent in the combination group was deployed first and then followed by a Fluency coaxially within it, which was opposite with our study. Currently, there is no consensus about the order of placing these two stents. At this point, the technique differs depending on the operators. In the present study, we placed Fluency stent firstly due to its good visibility and maneuverability, which is better to get an ideal position during the operation. Then the following bare stent could be fine-tuned according to the position of Fluency stent. Thirdly, there was no preference for the stent types among different operators in our study and they could go back and forth among stent types. On the contrary, in the former study a certain operator used a particular stent in clinical setting. Fourthly, we used different criteria of ultrasonography for TIPS shunt dysfunction. These details may determine the different results of outcomes between these two studies.

The TIPS dysfunction in uncovered stent is because of thrombosis and pseudointimal hyperplasia while in covered stent the shunt dysfunction is mainly related to suboptimal stent position [18, 19]. It has been suggested that placement of a stent with the cephalic end extended to the hepato-caval junction and the caudal end parallel to the vascular wall of the portal vein in a TIPS procedure can decrease the risk shunt dysfunction [10, 20]. As a result, to get the optimal stent position, the Viatorr stent placement requires accurate measurements so that a single Viatorr stent can extend from the portal vein access to the hepato-caval junction. In contrast, the technique of placing a combined bare/covered stent only require placing a Fluency first, followed by a bare stent across the parenchymal tract. In Saad’s study, 22% of TIPS creation with Viatorr device required an additional stent, which was significantly higher than that in our study (3%). Therefore, the impact of technique factor during the TIPS procedures on the outcomes needs more exploration.

The rebleeding rates in our study were comparable with recently published prospective RCTs, confirming that TIPS creation could significantly decrease the incidence rates of variceal rebleeding [2, 6, 16, 21]. Our results showed that the rebleeding rates were not different among groups. This may be explained by the similar shunt dysfunction among groups. Interestingly, we observed that in 27 all-cause rebleeding patients, 24 (89%) were related to portal vein hypertension. However, as showed in Fig. 2, the post-TIPS PPG in all patients decreased significantly. It seems that the post-TIPS PPG and the reduction of PPG were not associated with recurrent bleeding. This is consistent with the finding of a recent study that timing affects measurement of PPG after placement of TIPS in patients with portal hypertension [22]. It was suggested that the PPG measurement after immediate TIPS placement could not correctly predict the long-term prognosis, while PPG measured with the patient on stable clinical conditions was correlated with long term PPG and clinical outcomes [22]. Thus, repeat PPG measurement before patient discharge may be needed to determine reliable value.

The occurrence of HE is a devastating complication of TIPS. The reported post-TIPS HE rates using covered stent varied from 15 to 50% in different studies [23–25]. The incidence of post-TIPS HE in our patients was within the reported range. It is worth noting that we give diet education to patients after TIPS creation routinely in clinical practice. One of the most overriding concerns with the Fluency covered stent is the lack of 2 cm uncovered segment in the portal end, which may block the intrahepatic portal branches and decrease portal perfusion theoretically [7, 26]. However, in our study the incidences of post-TIPS HE were not different among groups. In our TIPS procedure, the distal covered portion of Fluency stents was placed into the left portal vein branch without entering the main portal vein in most cases. Thus, the hepatic perfusion from the portal vein blood flow was not significantly affected. This would help decrease the effect on liver function. In fact, previous studies have evaluated the outcomes of patients who underwent left vs right portal vein TIPS and verified that shunting the left portal vein could decrease the risk of HE and reduce TIPS dysfunction rates [20, 27, 28]. Our study provided another evidence for using the left portal vein in TIPS creation for variceal bleeding.

There were some limitations to the current study. First, although the baseline characteristics were comparable, the imbalanced sample size among groups might cause potential bias. And we failed to observe a statistically significant difference in shunt dysfunction rate though there is a trend towards Fluency group, which may due to the small sample size. In future, well-designed randomized controlled study with large sample size is needed to verify our results. Second, this study only included patients with indications for prevention of variceal bleeding. Further work is required to determine whether the Fluency covered stent and the generic bare stent/ Fluency covered stent is noninferior to Viatorr stent for TIPS placement in patients with other indications. Third, it was suggested that lamivudine could led to partial reduction of hepatic venous pressure gradient in patients with HBV-related cirrhosis. Long-term suppression of HBV by nucleoside analogs could prevent de novo onset or progression of esophageal/gastric varices [29]. Since most patients in the current cohort had HBV-related liver cirrhosis, the results should be interpreted cautiously for patients with other chronic liver diseases. Fourthly, we did not routinely perform endoscopy before and after TIPS creation in all patients. And thus, the endoscopic findings were not compared. Finally, the TIPS was performed only by experienced interventionalists in four tertiary hospitals. The findings need to be confirmed in more common clinical settings.

## Conclusion

Our study suggested that there was no significant difference of clinical outcomes among Viatorr stent, Fluency covered stent and generic bare stent/Fluency covered stent combination. Fluency covered stent and the generic bare stent/Fluency covered stent was not an unreasonable alternative to the Viatorr stent for TIPS creation.

## Supplementary Information

Supplementary Material 1: Supplementary Figure 1. Kaplan-Meier curves according to treatment group. Actuarial probability of (A) shunt dysfunction, (B) recurrent bleeding from any source, (C) overt hepatic encephalopathy and (D) death. Abbreviations: CI, confidence interval, HE, hepatic encephalopathy; HR, hazard ratio.

Supplementary Material 2: Supplementary Figure 2. Post-hoc competing risk analysis of outcomes with death and liver transplantation being the competing events. The cumulative incidence of (A) shunt dysfunction, (B) recurrent bleeding from any source, (C) overt hepatic encephalopathy, and (D) death. Abbreviations: CI, confidence interval, HE, hepatic encephalopathy; sHR, subdistribution hazard ratio.

Supplementary Material 3: Supplementary Figure 3. Univariate analysis of factors associated with shunt dysfunction. Abbreviations: HBV, hepatitis B virus; INR, international normalization ratio;  MELD, Model for End-Stage Liver Disease; PPG, portacaval pressure gradient; TIPS, transjugular intrahepatic portosystemic shunt.

Supplementary Material 4: Supplementary Figure 4. Forest plots showing the multivariate analysis of factors associated with outcome after TIPS. Forest plots indicating the multivariate analysis of factors associated with (A) shunt dysfunction, (B) all-cause rebleeding, (C) overt hepatic encephalopathy and (D) all-cause death after TIPSS. Hazard ratios are derived from multivariate Cox regression models, with 95% confidence intervals and P values for shunt dysfunction, recurrent bleeding, overt hepatic encephalopathy and death. Age (years), creatinine (mg/dL), sodium (mmol/L), MELD score (points), INR, and post-TIPS PPG (mmHg) were introduced into the multivariate Cox models as continuous variables. Abbreviations: HBV, hepatitis B virus; INR, international normalization ratio; MELD, Model for End-Stage Liver Disease; PPG, portacaval pressure gradient; TIPS, transjugular intrahepatic portosystemic shunt.

Supplementary Material 5: Supplementary Figure 5. Univariate analysis of factors associated with all-cause rebleeding. Abbreviations: HBV, hepatitis B virus; INR, international normalization ratio;  MELD, Model for End-Stage Liver Disease; PPG, portacaval pressure gradient; TIPS, transjugular intrahepatic portosystemic shunt.

Supplementary Material 6: Supplementary Figure 6. Univariate analysis of factors associated with overt hepatic encephalopathy. Abbreviations: HBV, hepatitis B virus; INR, international normalization ratio;  MELD, Model for End-Stage Liver Disease; PPG, portacaval pressure gradient; TIPS, transjugular intrahepatic portosystemic shunt.

Supplementary Material 7: Supplementary Figure 7. Univariate analysis of factors associated with death. Abbreviations: HBV, hepatitis B virus; INR, international normalization ratio;  MELD, Model for End-Stage Liver Disease; PPG, portacaval pressure gradient; TIPS, transjugular intrahepatic portosystemic shunt.

Supplementary Material 8: Supplementary Figure 8. Box plots showing the liver function variations. Box plots showing the variations of (A) albumin, (B) bilirubin, (C) creatinine, and (D) MELD score values during follow-up stratified by treatment. Abbreviations: MELD, Model for End-Stage Liver Disease.

## Data Availability

The datasets used and/or analyzed during the current study are available from the corresponding author on reasonable request.

## Abbreviations

TIPS: Transjugular intrahepatic portosystemic shunt
PTFE: Polytetrafluoroethylene
HE: Hepatic encephalopathy
RCT: Randomized controlled trial
IVC: Inferior vena cava
IQR: Interquartile range
OHE: Overt hepatic encephalopathy
HR: Hazard ratio
MELD: Model for End-Stage Liver Disease
