# Supplementary figures and images for: Comparison of outcomes for transjugular intrahepatic portosystemic shunt creation: Viatorr versus Fluency versus a bare stent/Fluency stent combination
Source: CVIR Endovasc. 2024 Oct 18;7:76. doi: 10.1186/s42155-024-00489-9 (PMC11489360; doi:10.1186/s42155-024-00489-9)

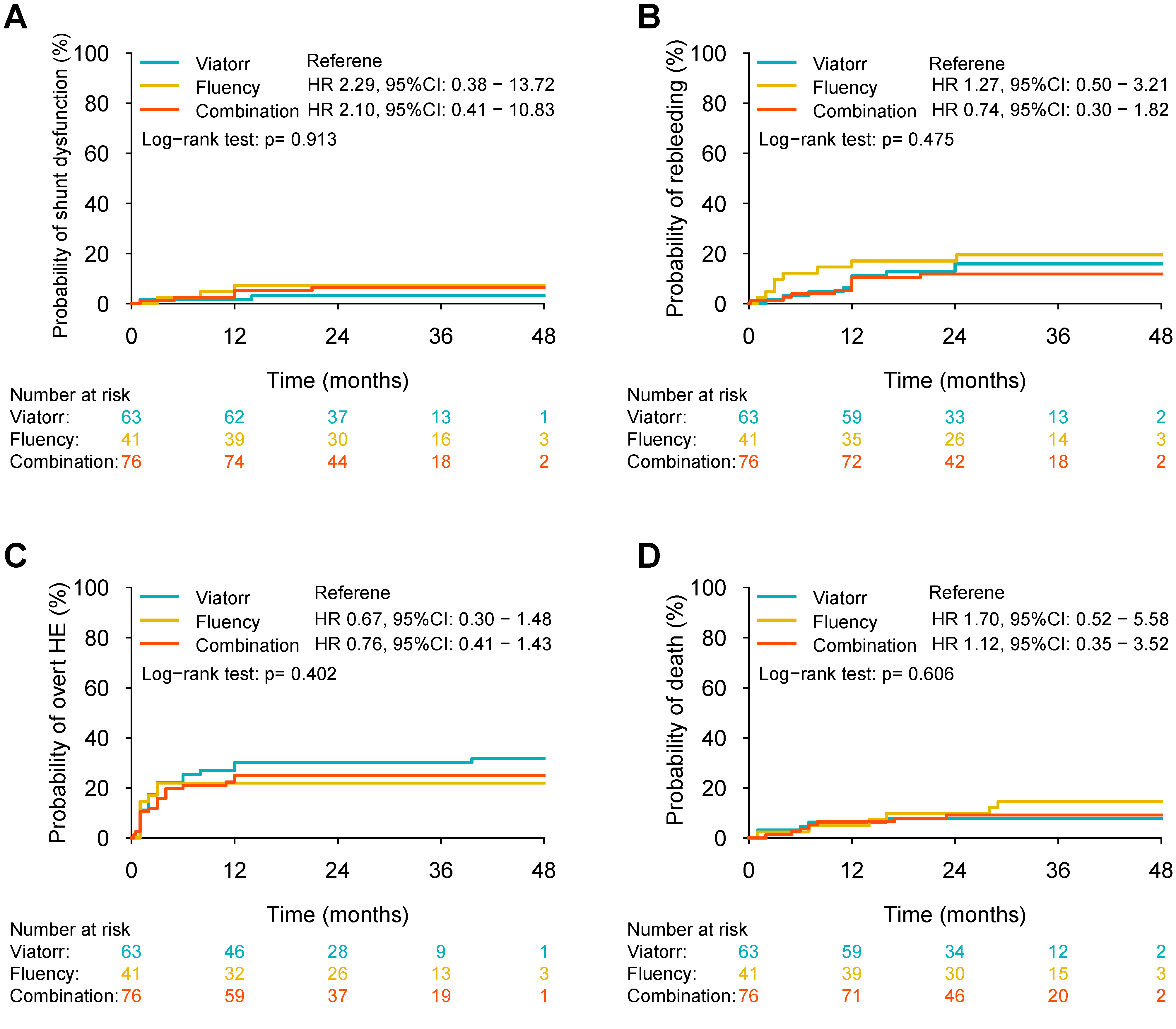

Supplement: Supplementary file 1 — Supplementary Material 1: Supplementary Figure 1. Kaplan-Meier curves according to treatment group. Actuarial probability of (A) shunt dysfunction, (B) recurrent bleeding from any source, (C) overt hepatic encephalopathy and (D) death. Abbreviations: CI, confidence interval, HE, hepatic encephalopathy; HR, hazard ratio. [file 42155_2024_489_MOESM1_ESM.tiff]

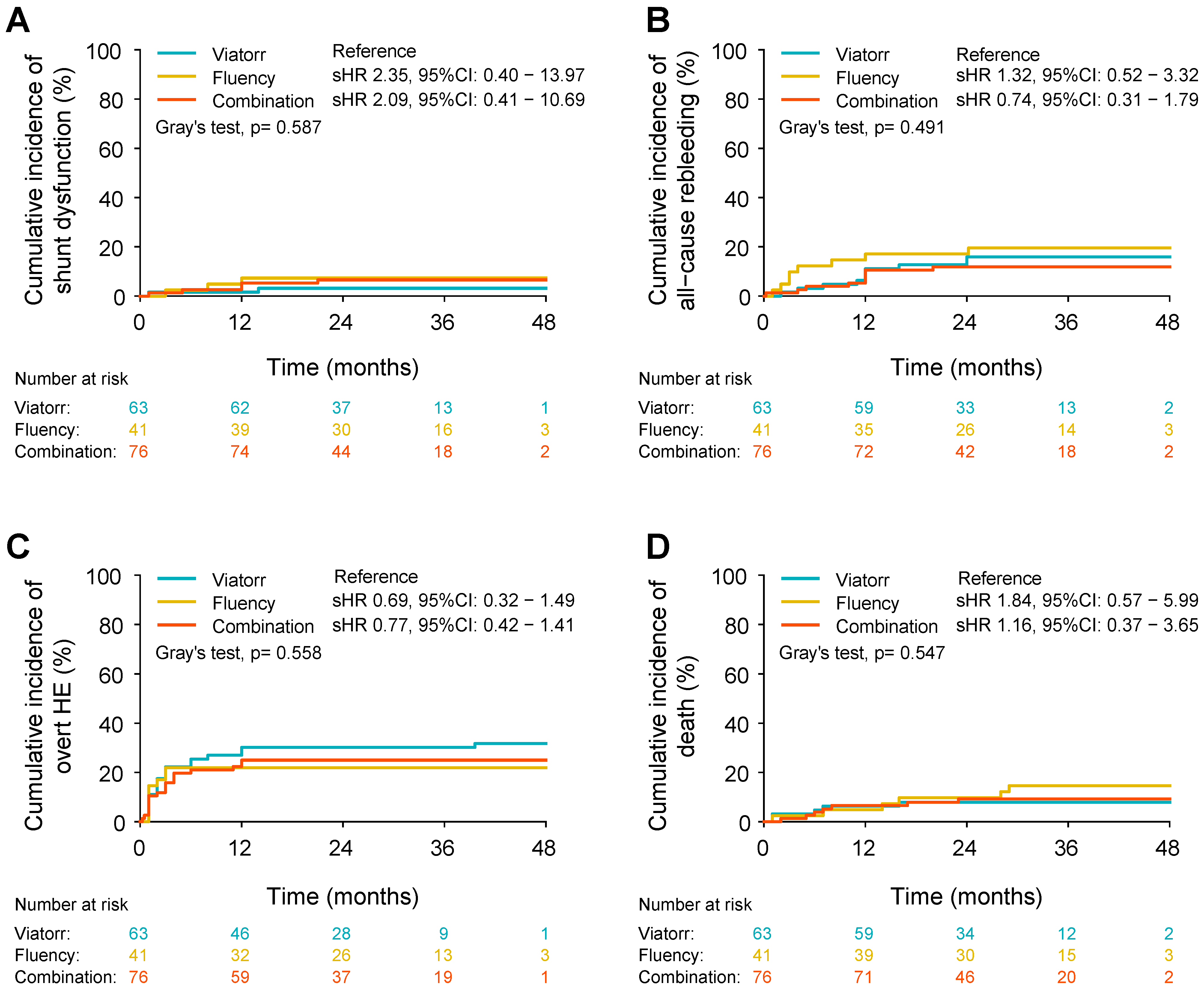

Supplement: Supplementary file 2 — Supplementary Material 2: Supplementary Figure 2. Post-hoc competing risk analysis of outcomes with death and liver transplantation being the competing events. The cumulative incidence of (A) shunt dysfunction, (B) recurrent bleeding from any source, (C) overt hepatic encephalopathy, and (D) death. Abbreviations: CI, confidence interval, HE, hepatic encephalopathy; sHR, subdistribution hazard ratio. [file 42155_2024_489_MOESM2_ESM.tiff]

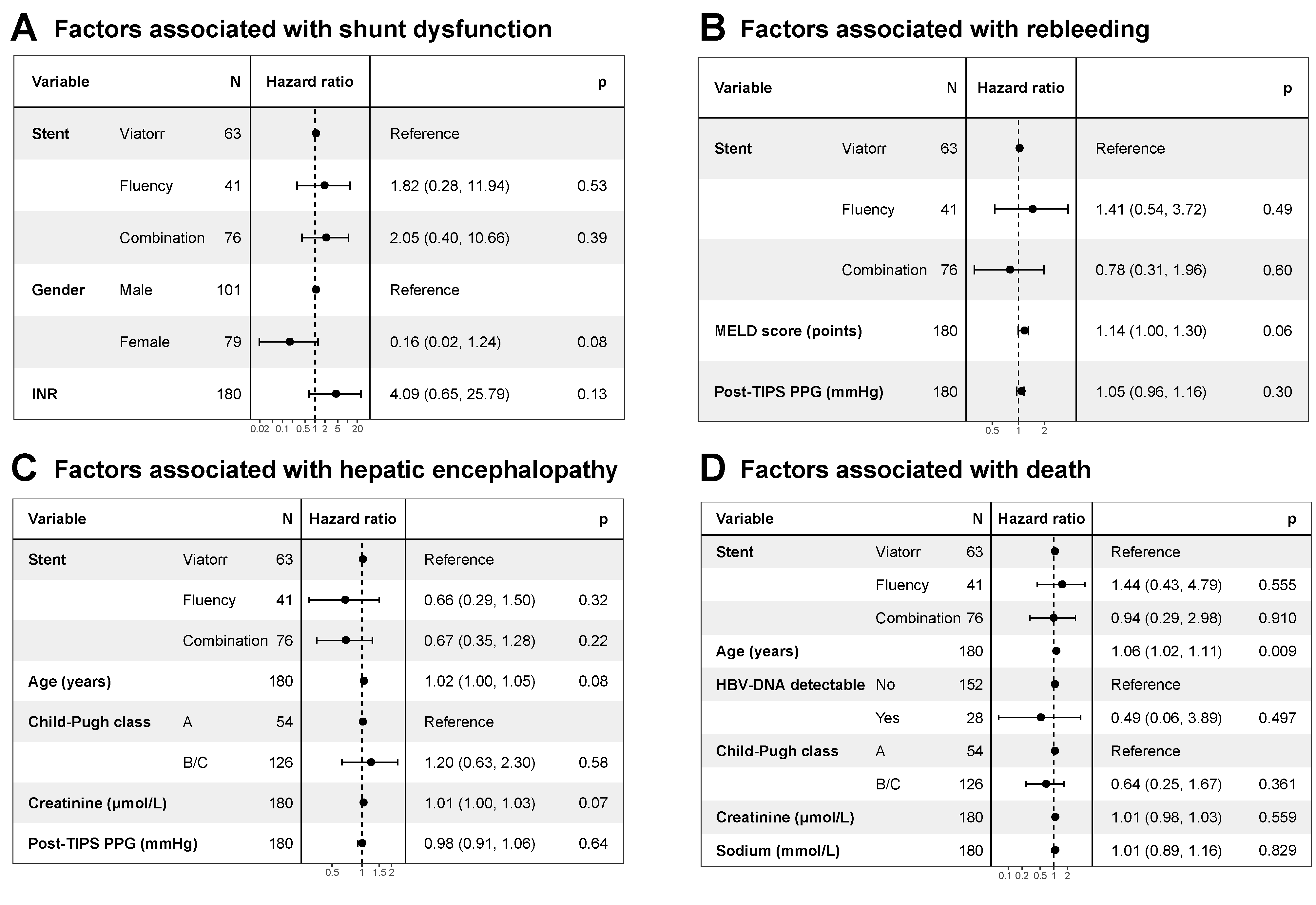

Supplement: Supplementary file 4 — Supplementary Material 4: Supplementary Figure 4. Forest plots showing the multivariate analysis of factors associated with outcome after TIPS. Forest plots indicating the multivariate analysis of factors associated with (A) shunt dysfunction, (B) all-cause rebleeding, (C) overt hepatic encephalopathy and (D) all-cause death after TIPSS. Hazard ratios are derived from multivariate Cox regression models, with 95% confidence intervals and P values for shunt dysfunction, recurrent bleeding, overt hepatic encephalopathy and death. Age (years), creatinine (mg/dL), sodium (mmol/L), MELD score (points), INR, and post-TIPS PPG (mmHg) were introduced into the multivariate Cox models as continuous variables. Abbreviations: HBV, hepatitis B virus; INR, international normalization ratio; MELD, Model for End-Stage Liver Disease; PPG, portacaval pressure gradient; TIPS, transjugular intrahepatic portosystemic shunt. [file 42155_2024_489_MOESM4_ESM.tiff]

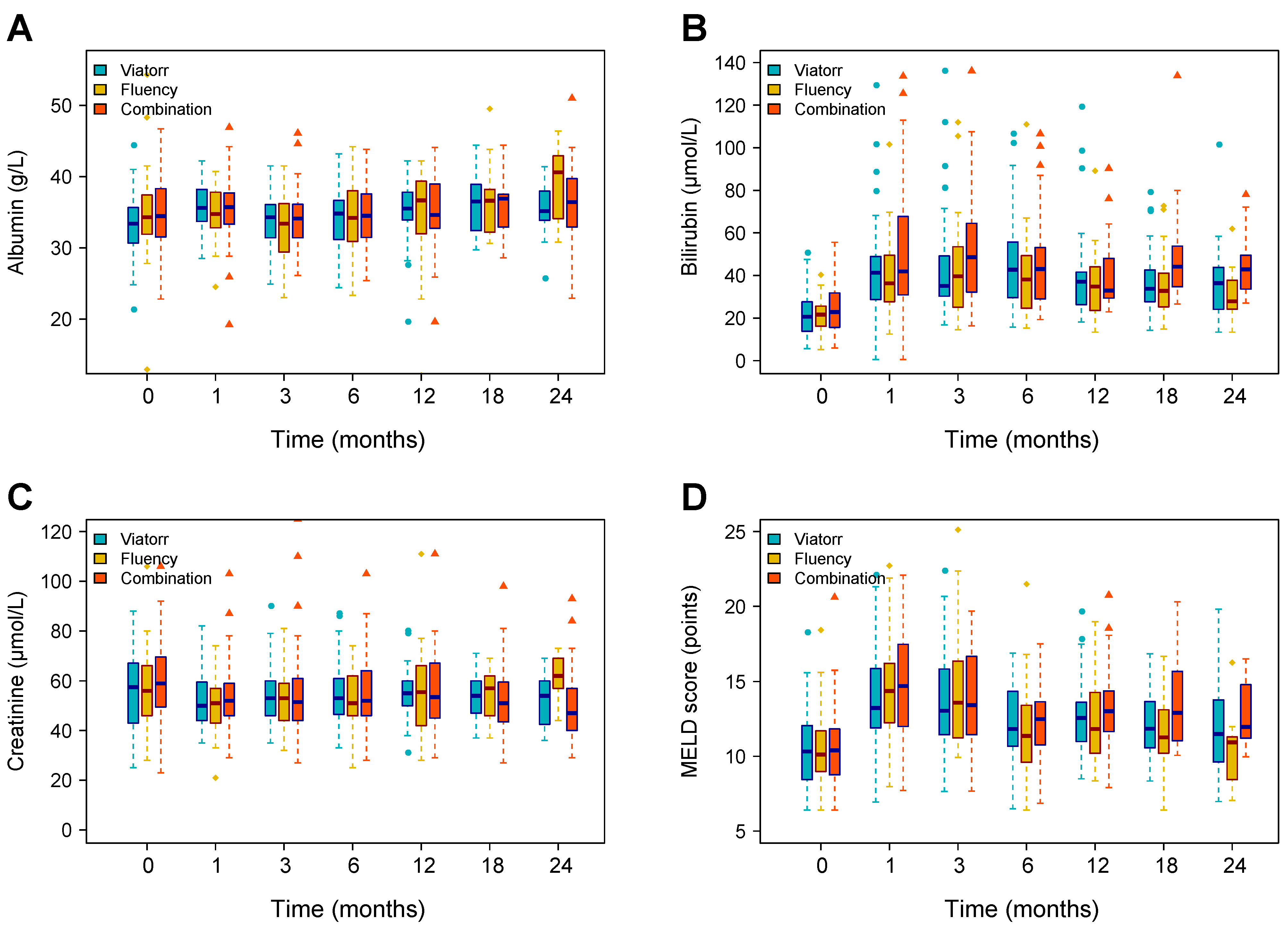

Supplement: Supplementary file 8 — Supplementary Material 8: Supplementary Figure 8. Box plots showing the liver function variations. Box plots showing the variations of (A) albumin, (B) bilirubin, (C) creatinine, and (D) MELD score values during follow-up stratified by treatment. Abbreviations: MELD, Model for End-Stage Liver Disease. [file 42155_2024_489_MOESM8_ESM.tiff]
